# “Family and society empowerment”: a content analysis of the needs of Iranian women who experience domestic violence during pregnancy: a qualitative study

**DOI:** 10.1186/s12905-023-02525-7

**Published:** 2023-07-12

**Authors:** Malikeh Amel Barez, Khadijeh Mirzaii Najmabadi, Robab Latifnejad Roudsari, Mojtaba Mosavi Bazaz, Raheleh Babazadeh

**Affiliations:** 1 Department of Midwifery, Faculty of Nursing and Midwifery, Mashhad Medical Sciences, Islamic Azad University, Mashhad, Iran; 2 Nursing and Midwifery Care Research Center, Mashhad University of Medical Sciences, Mashhad, Iran; 3 Department of Community Medicine, Faculty of Medicine, Mashhad University of Medical Sciences, Mashhad, Iran; 4 Department of Midwifery, School of Nursing and Midwifery, Mashhad University of Medical Sciences, Mashhad, Iran

**Keywords:** Empowerment, Need, Domestic violence, Pregnancy, Qualitative study

## Abstract

**Background:**

Domestic violence threatens maternal physical, psychological and emotional safety. Victim/survivor pregnant women required interventions based on their actual needs with the purpose of reducing domestic violence and its negative consequences. The present study aimed to explore the experiences of victimized Iranian pregnant women and identify their neglected needs.

**Methods:**

This qualitative descriptive study was performed from September 2019 to August 2021 in Mashhad, Iran. Semi-structured interviews with 14 women (8 pregnant and 6 after birth) who were the victims of domestic violence, and 11 key informants with various discipline specialties until the data saturation was achieved. Participants were selected through purposive sampling. Qualitative data were analyzed based on the conventional content analysis adopted by Graneheim & Lundman.

**Findings:**

The main theme emerging from the data analysis was “family and society empowerment” that implied the necessity of family, health system, legal, social and inter sectoral empowerment to reduce domestic violence during pregnancy. “Family and society empowerment” was comprised of three categories such as “need to empower couples to reduce domestic violence during pregnancy”, “demand for improved health care services”, and “need to strengthen inter-sectoral, legal and social supports”.

**Conclusion:**

Victim/survivor pregnant women experienced individual, interpersonal and inter sectoral needs. Family and society empowerment constituted the actual needs of victimized pregnant women. Awareness of policymakers and health system managers of these needs could be the basis for designing a supportive care program according to victim/survivor women’s actual needs. In addition to the educational and skill empowerment of couples, it is essential that supportive organizations cooperate with each other to provide integrated and coordinated services to victim/survivor pregnant women and strengthen and facilitate their access to supportive resources.

**Supplementary Information:**

The online version contains supplementary material available at 10.1186/s12905-023-02525-7.

## Introduction

Domestic violence is a severe social and public health problem, that affects more than one third of all women according to the World Health Organization’s global and regional estimates of violence against women in 2018 [1, 2]. Domestic violence is used frequently in place of intimate partner violence in the literature [3] which is physical, emotional, sexual abusive acts, and controlling behaviors performed by a present or previous intimate partner [4]. The prevalence of domestic violence during pregnancy was reported up to 20% [5] and 15–71% in low and middle income settings [6]. It is estimated that 19.3–94.5% of Iranian pregnant women experience domestic violence during their pregnancy [7, 8]. Pregnancy enforces important physical and psychological pressure on a woman, and when accompanied by other stress factors such as violence, they can adversely affect maternal and child health and increase maternal and neonatal morbidity and mortality [9, 10]. These perinatal effects include delayed entry to prenatal care, unplanned pregnancy, vaginal bleeding, miscarriage, preeclampsia, preterm labor, dystocia, low birth weight infants, inadequate antenatal weight gain, postpartum depression, and several mental problems [11–14].

Victim/survivor (V/S) pregnant women need interventions with the purpose of reducing domestic violence victimization such as screening, advocacy, supportive counseling service, and promoting safety behaviors [15]. Similarly, they need emotional, informational, financial, family and stakeholders supports, as well as legal assistance and counseling on how to manage marital difficulties [16]. Young pregnant women who are victims of violence need education, economic independence and lack of verification of the gender of the fetus during pregnancy, in addition to the treatment of alcohol-related disorder and the improvement of the spouse’s awareness of pregnancy [17]. Early identification of women experiencing domestic violence is the first step for screening and intervening to maintain their safety and well-being in many health systems [18–20]. Prenatal care is an opportunity for healthcare professionals to identify V/S pregnant women and consider proper counseling and intervention programs to protect the maternal and child health [21]. However, to tailor appropriate interventions and programs, it is required that V/S pregnant women’s needs to be identified during the perinatal timeframe to confirm adequate referrals and services to stop the cycle of violence [22]. These women require health care providers to be compassionate, ask them about their experiences of domestic violence and refer them to free supportive organizations. Also, they need their confidentiality and privacy to be maintained by the health care providers [23]. The V/S pregnant women require organized operational response to support them among the participating institutions, links between relevant supportive networks, and coordinated efforts to support them, along with formal training and awareness formation, perfect intervention guidelines, and changes in existing socio-cultural norms [24]. In low resource setting, it’s essential for V/S pregnant women to have food security, appropriate employment, and reduce mental health problems [25].

Despite the importance of respect to pregnant women and mothers in the Islamic rules, there are different kinds of domestic violence against pregnant women in Iran [26]. Iranian society is patriarchal and emphasizes men’s domination over women in the family. Women must obey their husbands, tolerate violence, and maintain the family and women who tolerate their husband’s abusive behavior are good women [27]. Based on the Iranian civil law if divorce occur the full custody of children after a certain age is being given to the father [27], and women prefer to stay in an abusive relationship than to divorce for different reasons such as fear of losing children, lack of social and legal support, and pressure of social judgment that stigmatize divorce [28]. Similarly, pregnant women prefer to stay in abusive relationships in Iran [29]. According to the specific Iranian cultural issue, pregnant women have few options to leave an abusive marital relationship. Therefore, there is a need for effective interventions to maintain maternal safety [20, 29]. The results of the study by Nayebi Nia (2019), the reproductive health needs of the victimized women included the need to encourage their self-care, to empower women against domestic violence, to provide a safe sexual life, to improve the capacity of the reproductive health system in support of victimized women, and to train men to be involved in reproductive health issues [30].

The high prevalence and the adverse consequences of perinatal domestic violence necessitate developing effective interventions based on the actual needs of victim/survivor pregnant women. Understanding the actual and unique needs of V/S pregnant women is crucial to develop evidence based domestic violence prevention strategies and programs. Qualitative study is essential to achieve a deeper understanding of the V/S women’s neglected needs during pregnancy and postpartum period. Despite the high prevalence of domestic violence during pregnancy in Iran, there is no evidence exist to explain the victim/survivor pregnant women’s actual needs. Therefore, the aim of this qualitative study was to explore the experiences of victimized Iranian pregnant women and identify their neglected needs.

## Methods

### Design

Qualitative description was used to design this study to gain deeper insight into pregnant women experiences in relation to a sensitive topic of domestic violence. Qualitative description follows the principles of qualitative research and is the method of choice when straight descriptions of phenomena are desired. It is well suited for “who”, *“*what” and *“*where” questions about human behavior, motives, and views [31]. It is a logical set of strategies for conceptual orientation, sampling, data construction, analysis, and reporting by which health science researchers plan to use an interpretive descriptive approach to develop knowledge about human wellbeing and disease [32]. The research paradigm was constructivist [33].

### Setting and participants

The study was accomplished in Mashhad, the second most populous city in Iran with total fertility rate of 1.9–2.1 births per woman [34] from September 2019 to August 2021. Urban health centers, obstetrics and gynecology departments of referral hospitals, midwifery counseling centers, forensic medicine center, and provincial social welfare center were used as the study settings.

Purposive sampling based on the maximum variation of mothers’ age (19–41), occupation status, education level, parity, wanted or unwanted pregnancy, gestational age and HITS (Hurt, Insult, Threaten and Scream) domestic violence positive screening score (11–20) was used to select the appropriate participants and key informants [35]. The inclusion criteria were pregnant or postpartum women with the self-reported experience of positive perinatal domestic violence through individual interviews. Exclusion criteria were substance abuse and clinically diagnosed physical and mental illnesses that prevented women from participation in the study.

### Data collection

Individual semi-structured in depth interviews were conducted with 14 women who experienced domestic violence during pregnancy and until one year postpartum period [36–39] and 11 female key informants who were specialist in different related disciplines comprising of reproductive health, midwifery, psychology, forensic medicine, social working, law, and sociology. Participants’ demographic data was achieved by the first author at the beginning of each interview. Some of the key informants’ interviews were conducted by telephone due to the outbreak of Covid-19 and home quarantine. Participants’ profiles are shown in Tables 1 and 2.

**Table 1:** The profile of victimized women participated in the study

participant	Age of woman/ husband	Education of woman /husband	Woman’s job	Husband’s job	Gestational age	HITS score*
1	37/ 53	8years/ 8 years	House wife	Retired	37w	16
2	22/ 29	7 years/ diploma	House wife	factory worker	39w	12
3	29/ 30	7 years/ diploma	House wife	Unemployed	40w	11
4	19/ 30	Diploma/ Associate Degree	House wife	factory worker	35w	17
5	25/ 25	Illiterate/ 6years	House wife	factory worker	10 h postpartum	12
6	41/ 47	Diploma/ 6 years	House wife	Sales Manager	45 days postpartum	16
7	36/ 31	Diploma/ 6 years	House wife	Driver	17w	18
8	24/ 28	Diploma/ diploma	House wife	Factory worker	20w	14
9	28/ 26	Diploma/ 6 years	Employed	Private business	8w	20
10	36/ 35	Doctorate/ master degree	Factory production manager	Factory production manager	1 year after birth	12
11	36/ 40	Master degree/ diploma	Engineer	Self employed	1 year after birth	12
12	26/ 31	Master degree/ doctorate	University teacher	Doctor	39w	12
13	36/ 40	Both Bachler degree	Employer	Self employed	1 year after birth	12
14	25/30	Both Bachler degree	House wife	employee	1 year after birth	18

* The HITS scale is a paper-and-pencil instrument that was comprised of the following four items: “How often does your partner: physically hurt you, insult you or talk down to you, threaten you with harm, and scream or curse at you?” Patients responded to each of these items with a 5-point frequency format: never, rarely, sometimes, fairly often, and frequently. Score values could range from a minimum of 4 to a maximum of 20 (35)

**Table 2:** The profile of the female key informants

Participant	Age	Education	Field of study	Work experience (years)	Job position
15	48	Bachelor’s degree	Midwifery education	23	Responsible midwife of health base
16	35	Bachelor degree	Counseling	12	Social Emergency Supervisor
17	52	Master’s degree	Sociology	25	Expert in charge of social welfare in the province
18	45	Master’s degree	Counseling	10	Consultant voice and In-person counseling center supervisor
19	50	Specialty in Medicine	Forensic medicine	15	Forensic expert
20	48	Master’s degree	Midwifery education	26	Responsible midwife, Midwife of the maternity clinic
21	35	Doctor of Philosophy	health psychology	10	Psychiatrist of the Comprehensive Health Center
22	38	Master’s degree	Criminal Law and Criminology	15	Lawyer and women’s activist
23	37	Doctor of Philosophy	Reproductive health	14	University Assistant Professor
24	47	Bachelor’s degree	Social work	23	Hospital social worker
25	48	Master’s degree	Maternal and child health	25	Director of the midwifery department, University instructor

The data were collected by the first author with experience of qualitative study and 24 years working experience in the field of reproductive health and midwifery education. All of the interviews were conducted in a separate room and suitable time for the participants to ensure their privacy and confidentiality. The interviews were audio recorded and observational notes and field notes were taken. Data saturation was achieved following 23 interviews. For data saturation assurance, two further interviews were done, which resulted in no new data. The interviews lasted between 45 and 90 min. Semi-structured interviews are used when the researcher has a list of general topics or questions to cover in the interview [33]. In the present study an interview guide with open ended questions was used to explore the actual needs of victim/survivor pregnant women as follows: “please describe your experience of domestic violence during the perinatal period?“, “Please describe your actual needs for reducing domestic violence”, “what do you expect of the health care system to provide for victimized pregnant women?“, “Which organization can help you to deal with domestic violence?“ and followed by probes to attain the additional information and descriptions.

### Data analysis

Conventional content analysis was used to answer the research question. The data were analyzed simultaneous during data collection according to the Graneheim and Lundman approach which allows the researchers to examine individual experiences and shows conflicting opinions and unsolved issues regarding meaning and use of concepts, procedures and interpretation via MAXQDA software (version 10, VERBI Software, Berlin, Germany) [29, 40–42]. After each interview the first researcher listened to it several times to get a general understanding of the content and then transcribed the interview verbatim and read it repeatedly to gain a deeper understanding of the data. The text of each interview was divided into meaning units. The meaning units were then condensed, summarized and coded. Codes were compared according to the similarities and differences and categorized into subcategories and categories and finally the theme developed.

### Ethical consideration

According to the Helsinki Declaration, the participants were fully informed about the purpose and benefits of the study as well as their voluntary participation [43]. They were reassured that they could withdraw from the study any time without prejudice. Written informed consent was obtained from all the participants and illiterate’s legal guardian. If any of the questions caused distress for the participants, the interview was stopped and after a while, and by the participant’s permission, it was continued. The participants’ privacy and the confidentiality of data were maintained. Each participant was given a hypothetical code and name to keep their information confidential. At the end of the interviews, necessary information about the existing supportive services for victim/survivor women was given to the participants. The study was approved by the Local Research Ethics Committee of Mashhad University of Medical Sciences and the Ethics Code was IR.MUMS.NURSE.REC.1398.026.

### Trustworthiness

Credibility, dependability, confirmability and transferability were used to confirm the rigor and the trustworthiness of the data [44]. To maintain the credibility of the results, three expert supervisors (Professors in Reproductive Health) reviewed and confirmed the processes of data collection, data analyzing and interpreting the results. Similarly, purposive sampling with maximum variation and appropriate participants and key informants were used. For dependability, the correctness of data analysis was confirmed by three independent skilled researchers in qualitative research (Assistant professors in Reproductive Health). For confirmability, some of the transcripts, along with the codes and categories were provided to the main supervisor (Assistant professor in Reproductive Health) as well as two other faculty members outside the field of the study, who were skilled in qualitative research and the process of analysis was confirmed. For transferability, the study characteristics such as the context of the research, participants, process of data collection and data analysis were explained in detail for further evaluation.

### Findings

The participants were 14 women (8 pregnant and 6 after birth) who were the victims of domestic violence, and 11 key informants with various discipline specialties. The victim/survivor women’s age were between 19 and 41 years and 30 years old in average. Domestic violence screening score (HITS score) ranged from 11 to 20. Educational levels of V/S women’s ranged from illiterate to doctor of philosophy degrees. Key informants were specialist in midwifery education (n:2), reproductive health (n:1), maternal and child health (n:1), counseling (n:2), forensic medicine (n:1), health psychology (n:1), sociology (n:1), law (n:1), and social work (n:1). None of the participants withdrew from the study.

In the present study 1479 codes, 14 subcategories, 3 categories, and 1 main theme emerged from data analysis. The “family and society empowerment” as the main theme was comprised of three categories including “need to empower couples to reduce domestic violence during pregnancy”, “demand for improved health care services”, and “need to strengthen inter sectoral, legal and social supports”. A more detailed presentation of the results including the main theme, three categories and related subcategories, are presented in Table 3.

**Table 3:** Victim/survivor pregnant women’s neglected needs

Sub categories	Categories	Theme
Need to enhance couples’ awareness	Need to empower couples to reduce domestic violence during pregnancy	Family and society empowerment
Necessity of improving couples’ life skills		
Enabling women’s economic empowerment		
Demand for preserving individual values		
Health care system informational and skills empowerment	Demand for improved health care services	
Professional ethics requirements		
Service demand for psychological care		
Need to maternity care improvement		
Investing for health care infrastructures		
Empowering the health education system		
Provision and strengthening inter-sectoral support	Need to strengthen inter sectoral, legal and social supports	
Reform the educational system		
Reform and implementation of women’s protection laws		
Need to receive social support		

### Main theme. Family and society empowerment

The main theme that emerged from the data analysis was family and society empowerment that implied the necessity of family, health system, legal, social and inter sectoral empowerment to reduce domestic violence during pregnancy.

### Category 1. Need to empower couples to reduce domestic violence during pregnancy

Empowering couples as promoting couple’s information, improving couples’ life skills, economic empowerment, and preserving individual values could reduce violence during pregnancy.

#### Need to enhance couples’ awareness

Enhancing couples’ awareness about individual rights, violence definition and different types of domestic violence along with promoting their awareness about pregnancy, childbirth, hormonal changes, physical and psychological changes following pregnancy and childbirth, and maternal and child care provide their empowerment and reduces violence. The following quotes reflected the importance of couples’ awareness:

*“…men should be trained about maternal changes during pregnancy, they should know about maternal physical, mental and sexual changes…” (Participant 10 − 1 year after birth)*.

#### Necessity of improving couples’ life skills

Learning life skills is one of the most important needs of couples to reduce domestic violence. Improving couples’ life skills such as anger control, interpersonal communication, self-care, problem solving, decision making, stress reduction, empathy, creative thinking, self-awareness, and critical thinking decreases perinatal violence. One victimized woman stated about the importance of learning life skills:

*“I need to know about life skills such as anger management and problem solving. We must learn more about life skills.“ (Participant 11 − 1 year after birth)*.

#### Enabling women’s economic empowerment

Learning self-employment skills, learning income-generating skills and couple career’s advancement reduce economic dependence of the mother on the spouse and partner violence, appropriately. One victimized woman stated the importance of maternal financial independence:

*“I must have a source of income for myself. If I have my own income and can manage my life economically, I don’t have to endure violence at any cost.“ (Participant 11 − 1 year after birth)*.

#### Demand for preserving individual values

Maternal belief in self-worth, love herself, demand for individual rights, learn self-esteem, and promotion skills and confidence preserved individual values and should be considered for victim/survivor pregnant women. The following statements confirm the importance of maternal individual values:

*“When I value myself as a mother, my husband values me too. If I value myself, I will not allow my husband to be violent.“ (Participant 13 − 1 year after birth)*.

According to the explained quotes, couple’s informational, life skills, economics, and legal empowerment could reduce domestic violence.

### Category 2. Demand for improved health care services

The health care system must be able to provide effective services for victim/survivor pregnant women. This capability would be achieved through their informational and ethical promotion, psychological and physical care promotion, improve the infrastructures, and empowering the health education system to provide services to V/S pregnant women.

#### Health care system informational and skills empowerment

Health care system empowering could be achieved by improving the informational capacity of health care providers in the field of domestic violence, their familiarity with relative laws and legal issues, and obtain the necessary information about supportive and referral centers for victim/survivor pregnant women. The following quote reflects the importance of health care providers’ informational empowerment:

*“The midwife must be trained for domestic violence counseling. She should be able to communicate well with victimized pregnant women and guide them correctly.“ (Key informant- participant 23- Assistant Professor in Reproductive health)*.

#### Professional ethics requirements

Learning professional ethics by health care providers, offer equitable and non-judgmental services for victim/survivor women, and respect the privacy and confidentiality of maternal information improve health system ethical empowerment. One victimized woman explained:

*“I need to be supported without judgment and blame during perinatal care, while keeping my information confidential.“ (Participant 14 − 1 year after birth)*.

#### Service demand for psychological care

Psychological counseling improvement could be achieved by providing effective psychological counseling for all pregnant women and specific psychological care for victim/survivor women. One victimized woman stated maternal requirement of psychological care:

*“I did not know that the health center has a psychologist who provides me free counseling and I can talk to her about the effective ways to deal with my husband’s violence. I needed psychological counseling.“ (Participant 14 − 1 year after birth)*.

#### Need to maternity care improvement

Improving the provision of care services was essential for victim/survivor pregnant women. Continuous midwifery care aimed at identifying and managing all kinds of violence during pregnancy and postpartum and carefully monitor the consequences of domestic violence on maternal and fetal health was essential for improving maternal physical care. One victimized pregnant woman pointed out the necessity of the presence of experienced health care personnel:

*“The midwife never asked me about the violence during perinatal care. Every time I went to the health center, she took care of me carelessly and quickly.“(Participant 1–37 weeks of gestation)*.

#### Investing for health care infrastructures

Empowering the health care system to provide effective services for victim/survivor pregnant women required infrastructure reforms such as developing a comprehensive protocol, reforming of the personnel structure, allocation of required resources and providing health care professionals’ physical and legal security. The following statements indicate the necessity of providing human resources for health care system to provide effective services for V/S pregnant women:

*“We do not have enough midwives in the health care system to provide adequate care for victimized pregnant women.“ (Key informant- participant 15- Responsible midwife of the health base)*.

#### Empowering the health education system

The midwives should be empowered through academic education and empowering curriculum planning. Similarly, it was essential to improve the practical skills of midwifery teachers and students. One victimized pregnant woman explained:

*“I don’t want the midwifery student to look after me. She cannot communicate properly.“ (Participant 3–40 weeks of gestation)*.

The health care system empowerment to provide effective informational, psychological, continuous perinatal care and adequate infrastructure services for victim/survivor pregnant women was very effective in reducing domestic violence during the perinatal period.

### Category 3. Need to strengthen inter-sectoral, legal and social supports

Strengthening inter-sectoral, legal and social support included providing and strengthening cross-sectoral support, reforming the education system, reforming and implementing women’s protection laws, and receiving social support.

#### Provision and strengthening inter-sectoral support

The Welfare Organization was one of the most important supportive organizations, which provided social emergency services, face-to-face and telephone counseling, and a safe house for victim/survivor women. Strengthening welfare services could provide effective supportive services for V/S pregnant women. The following statement confirm this:

*“Many times victimized mothers are imprisoned at home and cannot go out for perinatal care and psychological counseling. There should be a 24- hour- telephone counseling center so that they can call for psychological advice.“ (Key informant- participant 18- Consultant voice and In-person counseling center supervisor)*.

Threat to the pregnant women’s physical security forced them to go to forensic centers to document the domestic violence. Facilitate and accelerate the provision of forensic services was essential for victim/survivor pregnant women. One victimized pregnant woman explained the need for provision of forensic services:

*“I come to forensic medicine because of my wife’s physical violence, but I do not know what they will do here. I have been waiting for several hours and I am very tired.“ (Participant 8–20 weeks of gestation)*.

Victim/survivor pregnant women should be supported by the social worker and closely monitor their living conditions. In order to provide optimal services, it was essential to establish a direct link between social work and other supportive system and refine the process of providing social work services to V/S pregnant women. This quote reflects it:

*“In cases of domestic violence, it is necessary for a social worker to be present in the health centers along with the psychologist, to connect with the social environment and the living environment of the victimized mother.“(Key informant- participant 21- Psychiatrist of the Comprehensive Health Center)*.

Police should support the victim/survivor pregnant women in the case of going to police stations as well as establishing the possibility of health centers to contact the police in the case of domestic violence which requires legal action. This statement reflects it:

*“A mother who is physically victimized by her husband should get a forensic referral letter from the police station….“ (Key informant- participant 19- Forensic expert)*.

Establish communication between different organizations providing support services for victim/survivor women such as the health care system, welfare organization, social emergency, social work, forensic medicine, judicial and legal system, and police was essential to have inter sectoral supports and reduce domestic violence in pregnancy. One responsible midwife stated:

*“There is a need for more inter-sectoral cooperation. I should have the opportunity to refer the victimized mother to welfare or forensic medicine.“ (Key informant- participant 20- Responsible midwife, Midwife of the maternity clinic)*.

#### Reform the educational system

It was necessary to reform the educational system with the purpose of preventing violence against women such as creating a culture of violence denial through textbooks, considering self-awareness and consider life skill workshops at different educational levels from kindergarten. One *Responsible midwife of the health base* explained:

*“We should learn about violence in our school and university.“ (Key informant- participant 15- Responsible midwife of the health base)*.

#### Reform and implementation of women’s protection laws

The legal system needs to be reformed in order to provide effective services to victim/survivor women. These reforms include eliminate the context of gender discrimination laws, refinement and implementation of women’s protection bill and prohibition of violence against women, and modify the divorce and child custody law. The following statement indicate this:

*“Men allow themselves to be violent to the pregnant women because the law always protects them.“ (Participant 10 − 1 year after birth)*.

#### Need to receive social support

Community support for victim/survivor pregnant women in denying violence, supporting women entrepreneurship, and eliminate the pyramid of gender power in the workplace were essential to reduce violence against pregnant women. Similarly, the media had an important role in reducing and managing violence at the community level. The following statements confirm this:

*“Society should react to violence against pregnant women. Society should not remain silent in the face of violence against women.“ (Key informant- participant 23- Assistant Professor in Reproductive health)*.

Strengthening the related inter-sectoral, legal and social support had been very important in providing effective services to victim/survivor pregnant women and reducing domestic violence during the perinatal period.

## Discussion

This qualitative study investigated the neglected needs of victim/survivor pregnant women in Iran. These unique neglected needs include empowering couples to reduce domestic violence during pregnancy, demand for improved health care services, and strengthen inter sectoral, legal and social supports.

Victim/survivor pregnant women received little training during pregnancy that did not meet their educational needs at all. They need to be informational empowered in the field of violence. the result is similar to Deuba et al. (2016) study who emphasized on the female education to reduce violence against young pregnant women [17] and Sabri et al. (2015) who recommended developing awareness programs [45]. Due to the necessity of educating the spouse about violence, maternal changes during pregnancy and childbirth and maternal care during pregnancy and childbirth, one educational session should be dedicated for them during prenatal care. Raising husband’s awareness about pregnancy is aligned with Deuba study that highlighted the importance of increasing men’s awareness about pregnancy [17]. Similar to Nayebi Nia study, couples should be educated about reproductive health such as safe sexual life and recognition about male and female role in sex determination of fetus and men’s participation during prenatal care [30], so they would be empowered through education and their sexual autonomy was improved. They should be aware about domestic violence and adverse impacts of violence in pregnancy and maternal and fetal health which is aligned with previous studies [24, 46].

Mostly, victimized pregnant women had limited earning potential. They had employment complications. Usually they were unemployed and they needed to be helped by direct financial support, reduced dependency on their husbands and became financially independent. This result is similar to other studies that confirmed the importance of financial support on intimate partner violence [8, 47]. Victimized pregnant women with economic difficulties faced dietary restrictions that prevented them from getting the nutrients they needed during pregnancy. They needed food assistance and expected the health care system or social worker to help them. The result is aligned with Field study [25].

Preserve individual values such as women’s rights, maternal self-esteem, and maternal self-awareness should be considered as one of the victim/survivor pregnant woman needs. This result is consistent with the previous study that confirmed urgent attention to maternal legal rights related to violence [48].

Victimized pregnant women confronted with several mental health problems which could be detected through violence screening session. They needed supportive psychological counseling services based on maternal self-care, advocacy, promoting safety behaviors and safety planning according to domestic violence’s risk profile. Their marital relationship should be evaluated and offered counseling on how to handle marital difficulties. These results are similar to other studies that showed the association of mental illness and intimate partner violence during the antenatal period and the need for care programs addressing mental health [15, 16].

Prenatal care provides a good opportunity for victim/survivor pregnant women to attend the health centers. Empowering the health care system toward domestic violence to provide effective services for pregnant women was very important. Midwives and other health care providers had inadequate knowledge and skills due to lack of academic training in the field of domestic violence. They should be provided with high quality training, continuous and permanent education and professional supports to respond to domestic violence more effectively. These results are similar to previous studies that confirmed the lack of formal training for health care providers in the field of domestic violence [49–51]. Health care professionals identifying the signs of domestic violence encouraged V/S pregnant women to speak out about their experiences. Midwives should know how and when to talk about violence and how to listen to V/S women’s challenging answers. They should ask about violence in a sensitive, kind, nonjudgmental and confidential way. Also, they should be trained in how to address domestic violence in prenatal care and effective screening for violence while this screening requires more than just asking a question. The results are similar to other studies that emphasized the importance of health care professional skill training in the field of domestic violence [20, 47, 49, 50, 52, 53]. According to the results midwives should be trained to strengthen their interpersonal skills to have a good relationship with V/S pregnant women. They should maintain women’s privacy and confidentiality during the perinatal care as well as having enough time to offer to each pregnant woman during prenatal visits. These results are similar to other studies that described these aspects of professional ethics [20, 23, 48, 50].

Usually V/S pregnant women had inadequate prenatal care and confronted with a poorly developed referral system. They needed continuity of care during pregnancy, childbirth and postpartum, and routine enquiry of domestic violence in antenatal care. Health care providers should provide adequate care for them and their child as well as free referral supportive services which are aligned with previous studies [23, 49, 50, 54].

To provide effective supportive services to victim/survivor pregnant women, it was necessary to improve health care deficiencies in resources and its infrastructures. Lack of time, lack of staff, and lack of safe environments for violence screening were some of resources deficiencies which should consider to be reformed. This results are aligned with previous studies that supported the requirement of health care safe screening environment and adequate time and individualization [49, 50].

Due to lack of effective intervention programs in the field of perinatal domestic violence, it was essential to develop clear intervention guideline for all health care professionals in order to identifying and managing perinatal domestic violence to eliminate violence. This result is similar to other studies that emphasized the necessity of intervention protocol development for the health care professionals [24, 55, 56].

Providing supportive professional networks for the victim/survivor pregnant women, easy access to supportive system, strong referral systems and social support were related to reduce risk of undergoing domestic violence during pregnancy. Barriers of supportive resource utilization should be eliminated and pregnant women should have the chance to talk about their experiences and receive support. The results are similar to previous studies [45, 50, 57–59].

The Welfare Organization in Iran is one of the important supportive organizations, which provide telephone counseling for violence exposed women. Because these women were often isolated and could not assess direct and face to face supportive care, telephone counselling was one of the essential intervention for them. This result is similar to Kuhn study that emphasized on telephone and mobile intervention for intimate partner violence exposed women [60].

Victimized pregnant women in Iran do not receive protection in the judiciary system. There was no supportive law to protect women from violence in pregnancy. The legal system needs to be reformed in order to provide effective services to victim/survivor pregnant women. They needed to offer legal assistance in child custody cases for V/S pregnant women who preferred to stay in their marriages. This result is align with Sigalla et al. study study that supported considering legal assistance in child custody [16].

Some victim/survivor pregnant women needed to get help from the police and criminal justice system, especially when they confronted with severe physical violence, therefore it was essential to review and reform the protocol of these support systems according to maternal needs. This result is consistent with Sabri et al. (2015) study [45] but different from Gashaw et al. (2019) study with the consequence of reluctance police involving [24].

Victimized pregnant women needed to have social support. They needed changes in several discriminatory socio-cultural norms and cultural taboos such as shame, fear and judgment in the field of violence, divorce and remarriage. The society should be aware about violence and use its capacity to eliminate domestic violence in pregnancy. This result is similar to previous studies [16, 24]. The media had an important role on awareness and sensitization of the society about different types of violence against women and reduce violence in the society. Similar to Humphreys et al. (2011) study, the results of present study demonstrated the benefit of brief video on victim/survivor pregnant women’s awareness and disclosure of violence with the health care professionals [61].

Several organizations in Iran, including the health care system, the welfare organization, forensic medicine, social work and legal system supported victim/survivor pregnant women. But these organizations did not have a defined relationship with each other, and this was a big gap in providing services to these women. One of the most important needs of V/S pregnant women was the cooperation of these supportive organizations with each other to be able to provide integrated and coordinated services to V/S pregnant women and strengthen access to supportive resources. Similar to Marquesa et al. (2017) research the result of the present study emphasized on strengthening inter sectoral action that was essential for V/S pregnant women [56, 62] which emphasized on the organized and structured link between relevant supportive organizations. In contrast to Deuba et al. (2016) study that emphasized on establishing separate organizations within their society to handle domestic violence against young pregnant women [17], the result of the present study considered cooperation of existing supportive organizations with each other to be able to provide integrated and coordinated services to V/S pregnant women and strengthen access to supportive resources without need to allocate a special organization to this issue. This can also be economically valuable, especially in countries with limited resources. The present study emphasized on establishing a direct link between social work and other supportive system and refinement the process of providing social work services as well as facilitate and accelerate the provision of forensic services for victim/survivor pregnant women which were not implicated in previous studies.

The results of present study increase understanding of Iranian pregnant women’s actual needs for managing domestic violence which could enable supportive systems to develop more effective interventions to reduce domestic violence. Empowering couples as well as the health care system to reduce violence during pregnancy and involve community participation and inter-sectoral support in domestic violence management were the basis for designing supportive care programs. Due to the lack of effective intervention programs in the field of domestic violence in pregnancy, it is essential to have a further research with the purpose of developing clear intervention guidelines and a plan of action for all supporting organizations to reduce domestic violence in pregnancy.

### Strength and limitations

In order to gain a deeper understanding into the neglected needs of victim/survivor pregnant women, the present research was conducted on both pregnant and postpartum women who had the experience of perinatal domestic violence. Given that the research team intends to design an action plan for V/S pregnant women, data collection through interviewing with key informants from various relevant specialties was another strength of this study.

The specific socio-cultural norms of Iran and the difficulty to obtain responses from the victim/survivor pregnant women considering the taboo of violence and its consequences were important limitations of this study that may lead to under reporting of actual domestic violence in pregnancy. Some of the interviews were conducted by telephone due to the outbreak of Covid-19 and home quarantine, which was another limitation of the present study.

### Implications for practice and research

The results of the present study suggest that several interventions are necessary to reduce domestic violence in pregnancy such as couples’ educational, psychological, and financial empowering along with improving and empowering health care system and strengthen inter sectoral, legal and social supports. Midwives and health care professionals have a fundamental role in detecting domestic violence and provide appropriate care during pregnancy for victim/survivor women because of their sensitivity and nearness to the pregnant women. Therefore, operational action plan and educational framework for midwives and health care professionals will be an essential step in attaining sustainable influence of maternal and child health.

## Conclusion

The findings of this qualitative study emphasized the neglected needs of victim/survivor pregnant women. Victimized pregnant women experienced individual, interpersonal and inter sectoral needs. Awareness of policymakers and health system managers of these needs could be the basis for designing a supportive care program according to V/S women’s actual needs. It is essential that supportive organizations cooperate with each other to provide integrated and coordinated services to V/S pregnant women and strengthen and facilitate maternal access to supportive resources.

## Electronic supplementary material

Below is the link to the electronic supplementary material.

Additional File: Participants interview guide

## Data Availability

The datasets generated and/or analysed during the current study are not publicly available due to the sensitive nature of the data and in order to maintain the anonymity and privacy of the participants, but are available from the corresponding author (babazadehr@mums.ac.ir) on reasonable request.
