# Supplementary material for: “Family and society empowerment”: a content analysis of the needs of Iranian women who experience domestic violence during pregnancy: a qualitative study
Source: BMC Womens Health. 2023 Jul 12;23:370. doi: 10.1186/s12905-023-02525-7 (PMC10339606; doi:10.1186/s12905-023-02525-7)
Supplement: Supplementary file 1 — Additional File: Participants interview guide [file 12905_2023_2525_MOESM1_ESM.docx]

**Participants interview guide**

Please describe your experience of domestic violence during the perinatal period?

please elaborate any needs of victimized pregnant women that you feel?

what do you expect of the health care system to provide for victimized pregnant women?

Please describe your actual needs for reducing domestic violence?

Which organization can help you to deal with domestic violence?
